# FSH receptor N680S genotype-guided gonadotropin choice increases cumulative pregnancy and live birth rates after *in vitro* fertilization

**DOI:** 10.3389/fendo.2025.1576090

**Published:** 2025-05-13

**Authors:** Ida Hjelmér, Mathilda Nilsson, Emir Henic, Piotr Jędrzejczak, Hannah Nenonen, Katarzyna Ozegowska, Aleksander Giwercman, Margareta Laczna Kitlinski, Yvonne Lundberg Giwercman

**Affiliations:** ^1^ Department of Translational Medicine, Lund University, Malmö, Sweden; ^2^ Scanian University Hospital Malmö, Reproductive Medicine Center, Malmö, Sweden; ^3^ Department of Cell Biology, Poznan University of Medical Sciences, Klinika Pastelova, Poznan, Poland; ^4^ Department of Infertility Diagnosis and Treatment, Poznan University of Medical Sciences, Poznan, Poland

**Keywords:** FSHR, IVF, polymorphism, infertility, gonadotropin, pregnancy, live birth

## Abstract

**Objective:**

This study aimed to compare cumulative [fresh and frozen embryo transfers from one ovarian stimulation (OS) cycle] pregnancy and live birth rates in women for whom the choice between recombinant FSH (rFSH) and urinary FSH (uFSH) for OS was linked to FSH receptor (FSHR) N680S genotype and compared these to non-genotyped controls.

**Methods:**

To define the optimal combination of FSH type and FSHR genotype, 475 women were allocated to either the rFSH group or to the uFSH group for OS. The number of aspirated oocytes, cumulative pregnancy rates, and live birth rates in the first OS cycle were determined. Subsequently, their FSHR N680S (rs6166) variant was analyzed. Clinical data were backed up by *in vitro* experiments, in which COS-1 cells were transfected with homozygous FSHR variants and stimulated with either uFSH or rFSH. cAMP was measured to evaluate receptor activity. Thereafter, a sub-cohort of 221 who received optimal FSH treatment in relation to their FSHR genotype was selected from the total cohort of 475 women. Cumulative pregnancy and live birth rates were compared between 991 non-genotyped controls and these 221 women. Binary logistic regression was used to explore the odds ratios (ORs) and 95% confidence intervals (CIs) for cumulative pregnancy and live birth rates in the first OS cycle among genotyped and optimally treated women, with the non-genotyped cohort set as the reference. Adjustment was made for age, body mass index, and method of fertilization.

**Results:**

The combined clinical and *in vitro* data indicated that uFSH was the optimal choice for FSHR N680S S-allele carriers, whereas rFSH was the hormone of choice for asparagine (NN) subjects. The sub-cohort consisting of uFSH-treated S-carriers together with rFSH-treated NN-carriers had a significantly higher chance of pregnancy (51% vs. 40%; OR: 1.40, 95% CI 1.12-1.75, p=0.003) and live birth (40% vs. 29%; OR: 1.55, 95% CI 1.23-1.96, p<0.001) compared to non-genotyped women, in whom the choice of hormone was based on a standard clinical evaluation.

**Conclusion:**

A significantly increased chance of pregnancy and live birth can be achieved by a genotype-guided approach. While the administration of uFSH should be the choice for S-carriers, rFSH is beneficial for NN-carrying women.

## Introduction

It is estimated that over 2.5 million annual hormonal cycles are completed globally each year and that approximately 500,000 babies are born after *in vitro* fertilization (IVF) (1–4). This number is expected to increase in the years to come due to postponed parenting in combination with the worldwide expansion of access to assisted reproduction.

Ovarian stimulation (OS) is the first crucial step in both standard IVF and intracytoplasmic sperm injection (ICSI). This is achieved with the gonadotropin follicle-stimulating hormone (FSH) for the growth of multiple follicles and, subsequently, with human chorionic gonadotropin (hCG), which is a luteinizing hormone (LH) agonist, for oocyte maturation. Given the availability of different FSH preparations, i.e., purified or highly purified urinary preparations (uFSH), and subtypes of recombinant FSH (rFSH), clinicians are faced with the question of which type of FSH to use to maximize the live birth rates (5). In addition to the type of FSH, genetic variants of the FSH receptor (FSHR) may play a role in the hormonal response. Several studies, including the largest meta-analysis to date, have shown that despite the same FSH doses and treatment durations, more oocytes are available for fertilization in women homozygous for asparagine (NN) at the FSHR N680S (rs6166) position compared to those with serine (SS) at the same position (6, 7). Notably, in these studies, the majority of patients were treated with rFSH. Recombinant FSH was also used when there was higher *in vitro* receptor activity for the NN as compared to the SS variant (8). However, data are sparse regarding the impact of the FSHR N680S polymorphism on uFSH effects. Lledo et al. (2013) (9) reported an almost 10% higher clinical pregnancy rates in SS-homozygous and NS-heterozygous, i.e., S-allele-carrying women, as compared to NN-homozygous women undergoing OS with uFSH. In this relatively small study, the difference was not statistically significant. However, in another study by the same group, significantly more oocytes and metaphase II mature oocytes (MII) were retrieved in FSHR N680S SS-homozygous women when given uFSH as compared to rFSH (10). In heterozygous women, however, more oocytes and MII were produced in response to rFSH. The authors speculated that the longer half-life of uFSH may explain the result observed in SS carriers. The presence of the more acidic isoforms of uFSH compared to the more basic rFSH molecule (11), and the maintenance of FSH action for a longer period of time may be beneficial for FSHR N680S SS-carrying women, as this particular group has previously been shown to be more resistant to FSH than carriers of other genotypes (12). They may, therefore, benefit from prolonged hormonal exposure. The importance of the diverse pharmacodynamic properties of the different FSH preparations has been stressed previously, but the clinical effect is not clear, and limited published data are available (13–16). Andersen et al. (2004) (11) have performed a meta-analysis comparing rFSH with uFSH. Overall, there was a lower total dose requirement and a shorter duration of treatment with rFSH, but rFSH generated a higher number of follicles and retrieved oocytes. There was no difference in the clinical pregnancy rate per patient, the clinical pregnancy rate per embryo transfer, or the ongoing pregnancy rate per embryo transfer, suggesting that rFSH was more potent in generating follicles and oocytes but not in pregnancy rates. However, except for the studies by Lledo et al. above, the FSHR N680S polymorphism has not been evaluated in this context.

Today, it is recommended that clinical decisions regarding the use of uFSH vs. rFSH should depend on availability, convenience, and cost (17), which is quite different from precision medicine, i.e., tailoring the treatment of patients based on their specific genetic characteristics, aiming to provide the best treatment options to each patient. To test the concept of precision medicine, and as previously suggested in the work of Lledo et al. (9, 10), we hypothesized that an increased cumulative (fresh and frozen embryo transfers from one OS cycle) pregnancy and clinical live birth rates (CLBR) could be achieved by applying FSHR N680S genotype-based selection of FSH for OS prior to IVF. Our objective was, therefore, to compare the cumulative pregnancy rates and CLBR in non-genotyped controls, in whom the choice between uFSH and rFSH had followed normal clinical routine, with a selected group of FSHR N680S genotyped women, i.e., S-allele-carrying women, to whom uFSH had been administered, and NN-homozygous women who had been treated with rFSH.

## Materials and methods

### Subjects

#### Study design

Study subjects were recruited among couples referred for IVF or ICSI at the Reproductive Medicine Centre, Skane University Hospital, Malmö, Sweden. The inclusion criteria were a) at least 12 months of unprotected intercourse without achieving pregnancy; b) female age <40 years; c) both ovaries functioning; d) body mass index (BMI) <30 kg/m^2^; e) normal ovulatory cycles (26–32 days); f) tubal factor, male factor, or unexplained infertility as an indication for treatment; and, g) the couple did not have a child together. The following exclusion criteria were applied: a) serum levels of anti-Müllerian hormone (AMH) <5 pmol/L, b) FSH>12 IU/L on cycle day 2-3, c) endometriosis, d) polycystic ovarian syndrome (PCOS), e) premature ovarian failure, f) current smoking, or g) male age >56 years.

**Figure 1:**
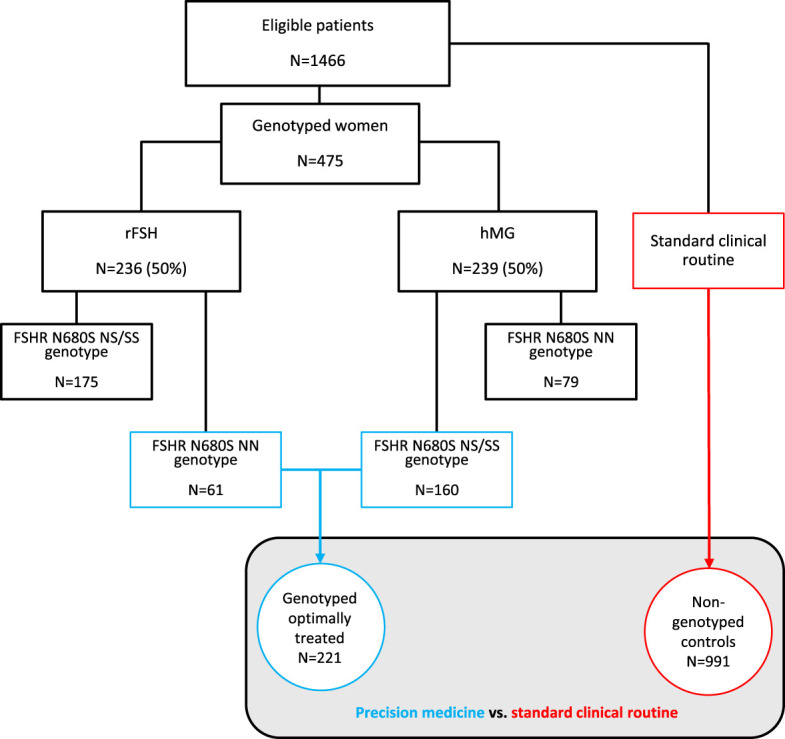
Flow chart of the included women. FSHR, follicle-stimulating hormone receptor; rFSH, recombinant follicle-stimulating hormone; uFSH, human menopausal gonadotropin.

The inclusion period was initiated in September 2016 and terminated in December 2020. During this period, 475 women in total were enrolled and followed until December 2021. The study design is described in **Figure 1**. Patient characteristics are presented in **Table 1**. The principle of alternating allocation using sealed envelopes was applied for randomizing the participants to either rFSH (Gonal-f®, Merck Serono S.A. Aubonne, Switzerland) or to uFSH (Menopur®, Ferring Pharmaceuticals, Saint-Prex, Switzerland). To avoid bias, the clinicians who were prescribing medication were neither involved in recruiting patients for the study, which was managed by a research nurse, nor aware of the participant’s genotype, which was determined afterward. Venous blood samples were drawn for DNA extraction and subsequent FSHR genotyping, whereafter normal clinical procedures were applied. Only the first OS cycle and the outcomes of the corresponding embryo transfers, both fresh and frozen, were included. The primary outcomes were cumulative pregnancy rates and CLBR for all treatments related to the first OS. The secondary outcome was the number of aspirated oocytes. The type of hormone given in relation to the FSHR N680S genotype is specified in **Table 2**.

**Table 1:** Background characteristics of the participants, whether genotyped or not, and type of FSH given to the genotyped women.

Background characteristics	All treatments			rFSH	uFSH	Genotyped + optimal FSH choice
	All (N=1466)	Genotyped (N=475)	Non-genotyped (N=991)	Genotyped (N=236)	Genotyped (N=239)	NN + rFSH / NS & SS + uFSH (N=221)
Age, mean (SD)	32 (3.9)	33 (3.8)	32 (3.9)	33 (3.7)	33 (3.9)	33 (3.8)
BMI, mean (SD)	24 (3.2)	24 (3.4)	24 (3.1)	24 (3.2)	24 (3.5)	24 (3.3)
AMH, median [range]	19 [5; 104]	19 [6; 81]	19 [5; 104	19 [6; 69]	20 [6; 81]	19 [6; 81]
Total dose, median [range]	1563 [13; 6150]	1688 [763; 4875]	1500 [13; 6150]	1525 [763; 4875]	1875 [784; 4875]	1688 [763; 4875]
Aspirated oocytes, median [range]	11 [0; 42]	11 [0; 37]	11 [1; 42]	11 [0; 37]	11 [0; 24]	11 [0; 37]
GQE, median [range]	2 [0; 12]	1 [0; 10]	2 [1; 12]	1 [0; 10]	1 [0; 8]	1 [1; 3]
IVF, n (%)	610 (53)	248 (53)	362 (52)	123 (53)	125 (54)	125 (58)
ICSI, n (%)	501 (43)	208 (45)	293 (42)	103 (44)	105 (45)	89 (41)
Micro combination*, n (%)	51 (4)	11 (2)	40 (6)	8 (3)	3 (1)	3 (1)

*Micro combination: 50% of oocytes fertilized using IVF and 50% by ICSI. AMH, Anti-Müllerian hormone; GQE, good quality embryo; IVF, *in vitro* fertilization; ICSI, intra cytoplasmic sperm injection; uFSH, urinary FSH; rFSH, recombinant FSH.

**Table 2:** Distribution of FSHR N680S in the study cohort.

Position	Variant	All (N = 475)	rFSH (N = 236)	uFSH (N = 239)
**FSHR N680S**	NN, n (%)	140 (29)	61 (26)	79 (33)
	NS, n (%)	237 (50)	123 (52)	114 (48)
	SS, n (%)	98 (21)	52 (22)	46 (19)

FSHR, follicle-stimulating hormone receptor; N, asparagine; S, serine; rFSH, recombinant FSH; uFSH, urinary FSH.

#### Genotyped and optimally treated cohort

Following the first part of the study, in which the 475 genotyped women were randomized to either uFSH or rFSH, and also based on the *in vitro* results (please see below), we defined a sub-cohort of 221 participants who had received optimal hormone OS according to their N680S genotype.

#### Non-genotyped control group

Women who refused genotyping or were not asked to participate in the above randomized study (n=991) were included as controls. These women were selected using the same inclusion criteria and were followed for the same time period as the genotyped cohort. Furthermore, for this group, CLBR and cumulative pregnancy rates for the first OS cycle were considered the primary outcomes.

The Swedish Ethical Review Authority (2016-467; 2023-05465-02; 2024-01055-02) approved the study. All genotyped women participated with written informed consent. Neither the patients nor the public were involved in the design, conduct, reporting, or dissemination of the aim of our research.

### Ovarian stimulation

In the genotyped group, 236 (50%) patients were treated with rFSH, whereas 239 (50%) patients received uFSH (**Figure 1**). GnRH antagonist protocols were used [Ganirelix®, Orgalutran, Organon (Sweden) Ltd., Stockholm, Sweden, or Fyremadel, SUN Pharmaceutical Industries Europe B.V., Hoofddorp, The Netherlands] in 98% of the patients, whereas a GnRH agonist (Synarela®, Nafarelin, Pfizer AB, Sollentuna, Sweden) was utilized in 2% of the subjects. Follicular development was monitored by vaginal ultrasound on stimulation days 6–8, and individual doses were adjusted if required. The median (range) total hormonal dose was higher in uFSH-stimulated women compared to rFSH-treated women [1,875.0 (250.0; 6,750.0) vs. 1,650.0 (31.0; 4875.0); **Table 1**]. Follicular maturation and oocyte release were triggered with 250 micrograms of hCG (Ovitrelle, Merck KGaA, Darmstadt, Germany) when ≥3 follicles reached 18 mm. Oocytes were aspirated 36 hours later. For luteal support, 3×200 mg/day progesterone (Lutinus, Ferring SA Hilding, Lausanne, Switzerland) was administered vaginally for 14 days. Meanwhile, oocytes were fertilized, either by ICSI or by standard IVF. The best embryo was transferred into the uterus, and an hCG test was done after 14 days. Pregnancy was confirmed by ultrasound at week 7–8.

The occurrence of severe ovarian hyperstimulation syndrome (OHSS) and miscarriage, defined as a spontaneous loss of a previous hCG-test confirmed pregnancy before gestational week 18, were also registered, but were not considered as study outcomes.

### Genotyping

DNA was extracted from peripheral leukocytes using the PureLink™Genomic DNA Mini Kit (Invitrogen, Life Technologies Corporation, Carlsbad, CA, United States) or the Quick-DNA™ Miniprep Kit (Zymo Research Corporation, Irvine, CA, United States). The FSHR N680S variants were analyzed after OS with the TaqMan allelic discrimination assay (AssayID C_2676874_10_; probes with FAM™ and VIC dyes, LifeTechnologies, Carlsbad, CA, United States) on a Bio-Rad CFX96 Real-Time PCR detection System (Bio-Rad, Stockholm, Sweden) in 25 µl reactions containing 10 ng DNA. Genotyping was undertaken at the Center for Translational Genomics (CTG; Faculty of Medicine, Lund University, Lund, Sweden). Randomly selected samples were directly sequenced by Sanger sequencing (LightRun sequencing, GATC Services, Eurofins Genomics, Ebersberg, Germany) for validation of the genotypes.

### FSHR activity *in vitro*

Extracellular cAMP activity was assessed as described previously (8). In short, COS-1 cells (ECACC, Salisbury, UK) were seeded into 12-well plates (150–000 cells/well) containing Dulbecco´s modified Eagle´s medium (DMEM; Gibco Invitrogen, Carlsbad, California, United States) supplemented with 10% fetal bovine serum (FBS; Biological Industries, Beit HaEmak, Israel) and 1% penicillin-streptomycin (5,000 U penicillin and 5 mg/ml streptomycin; Sigma-Aldrich, Stockholm, Sweden). The next day, cells were transfected with 1 µg pCMV6-XL5 plasmid (Origene Technologies Inc., Rockville, Maryland, United States) containing the genetic variants FSHR N680 or S680. An empty vector was used as a background control. After 24 h, the medium was replaced by phenol-red free and serum-free DMEM (Life Technologies, Stockholm, Sweden), and cells were incubated at 37°C, 5% CO_2_. One hour later, the cells were stimulated with 0, 1, 10, or 90 IU of uFSH (Menopur) or rFSH (Gonal-f) for 1h at 37°C, 5% CO_2_. After stimulation, the medium was aspirated and centrifuged for 20 min at 1000×g while the cells were lysed with RIPA buffer (Life Technologies, Stockholm, Sweden). Extracellular cAMP was assessed in the medium using a cAMP enzyme-linked immunosorbent assay kit (ENZO Life Sciences, Lausen, Switzerland). Total protein concentration was measured in the cell lysates with bicinchoninic acid protein assay reagent (Thermo Fischer Scientific Inc., Waltham, Massachusetts, United States) and used for normalization. All experiments were performed in duplicate and repeated three times.

### Statistical analyses

Statistical analyses were performed in R (version 4.2.2) (18) and SPSS software version 29 (SPSS, Inc., Chicago, IL, United States). Background data were described as mean and standard deviation for continuous variables, or, alternatively, median and range for non-normally distributed variables, and absolute and relative frequencies for categorical variables. The allele frequency of the FSHR N680S in comparison to another Caucasian infertile cohort (19) was evaluated using a chi-square test. Because the study was performed on candidate genes, no correction for mass significance was necessary (20).

#### Determination of optimal FSH treatment according to FSHR N680S genotype

As a first step, we determined in a clinical setup and *in vitro* whether the hormonal effects noted were FSHR N680S genotype-dependent or not.

#### Clinical setup

In the 475 genotyped women, logistic regression was applied to explore the odds ratio (OR) for the clinical outcomes (cumulative pregnancies and CLBR in the first OS cycle) following the use of uFSH and rFSH, with the latter as reference.

For the number of aspirated oocytes, linear regression was used instead, with a square root transformation of the outcome to meet the assumption of normality. Estimated marginal means (EMMs) were also computed. An adjustment for age and BMI was made.

Separately for NN- and S-carriers, optimal hormonal treatment (uFSH or rFSH) in the genotyped group was defined as a statistically significantly higher number of aspirated oocytes and/or higher cumulative pregnancy rates or CLBR.

#### *In vitro* data

Calculations of differences in cAMP production following stimulation with uFSH vs. rFSH in COS-1 cells expressing the FSHR N680 or S680 variant were assessed using a two-sample t-test assuming unequal variances.

#### CLBR and cumulative pregnancy rates in the genotyped group vs. controls

To clarify the primary aims of this study, binary logistic regression was applied to evaluate the difference in cumulative pregnancy rates and CLBR in the first OS cycle in the genotyped and optimally treated cohort vs. the non-genotyped control (**Figure 1**). The definition of optimal hormonal treatment was based on the above-described clinical and *in vitro* data.

Adjustments for female age and BMI (continuous variables) were done in all regression analyses. As a sensitivity analysis, an additional adjustment for fertilization method (IVF/ICSI) was performed.

A *post hoc* power calculation showed that with 221 subjects in the genotyped, optimally treated group and 991 individuals in the non-genotyped control group, we had 80% power (α=0.05) to detect a 33% relative increase in CLBR in the former group as compared to controls.

P<0.05 defined statistical significance. Since we hypothesized that rFSH treatment would be beneficial for NN carriers, whereas uFSH would be preferable for those with an S-allele, a one-sided p-test was used in all calculations where the effect of rFSH vs. uFSH was compared. In all other cases, a two-sided test was used.

## Results

### Background characteristics in the genotyped and the non-genotyped cohorts

There was no difference between non-genotyped women and those who were genotyped regarding mean (SD) age [32 years (3.9) vs. 33 years (3.8) and BMI 24 (3.1) kg/m^2^ vs. 24 (3.4) kg/m^2^], or median (range) AMH concentration [19 pmol/L (5; 104) vs. 19 pmol/L (6; 81)] (**Table 1**). The same was noted for the selection of fertilization method [IVF: 52% vs. 53%, ICSI: 45% vs. 42%, micro combination (50% oocytes fertilized by IVF and 50% by ICSI): 2% vs. 6%]. In the genotyped cohort, the FSHR N680S genotype distribution (**Table 2**) was similar to that in other Caucasian infertility cohorts (19).

### Optimal FSH treatment according to FSHR N680S genotype

#### Aspirated oocytes

The median number of retrieved oocytes was 11. More oocytes (33%) were retrieved in FSHR N680S NN women if treated with rFSH as compared to those given uFSH (EMMs: 12 vs. 9; adjusted β: -0.44, 95% CI -0.75; -0.14, p=0.004; **Table 3**). In S-carriers, no difference between the effects of the two types of hormones was observed.

**Table 3:** Estimated marginal means (EMMs) for aspirated oocytes with 95% confidence interval (95% CI), calculated for mean age and BMI in the adjusted analysis.

Hormone	FSHR N680S	Unadjusted	Adjusted
		EMMs (95% CI)	EMMs (95% CI)
rFSH	NN	12 (11; 14)**	12 (11; 14)**
uFSH	NN	9 (8; 11)**	9 (8; 11)**
rFSH	NS	10 (9; 12)*	10 (9; 12)*
uFSH	NS	11 (10; 12)*	10 (9; 12)*
rFSH	SS	10 (9; 12)*	10 (9; 12)*
uFSH	SS	10 (8; 11)*	10 (8; 11)*

FSHR, follicle-stimulating hormone receptor. N, asparagine. S, serine. rFSH, recombinant FSH. uFSH, urinary FSH. * - for the difference between rFSH and uFSH, p>0.05. ** - for the difference between rFSH and uFSH, p<0.05.

#### Cumulative pregnancy rates and CLBR in the first OS cycle

Among the 475 genotyped women, 375 women received fresh embryo transfers, and subsequently 126 women received frozen embryo transfers. In FSHR N680S S-carriers overall, uFSH treatment resulted in more pregnancies as compared to treatment with rFSH (53% vs. 43%; adjusted OR: 1.45, 95% CI 1.09–2.96, p=0.048; **Table 4**). Higher CLBR was also seen in uFSH-treated S carriers. However, this difference was not statistically significant (43% vs. 37%, adjusted OR: 1.29, 95% CI 0.82–2.02, p=0.133). In NN women, no differences between hormones regarding the chance of pregnancy or CLBR were evident (**Table 4**). The results did not change following the adjustment for fertilization method.

**Table 4:** Cumulative pregnancy rates and clinical live birth rates for the FSHR N680S-genotyped population (n=475).

Hormone	FSHR N680S	Cumulative pregnancy rate, n (%)	Clinical live birth rate
rFSH	NN	28 (46)*	21 (34)*
uFSH	NN	37 (47)*	30 (38)*
rFSH	NS/SS	76 (43)**	64 (37)*
uFSH	NS/SS	85 (53)**	68 (43)*

FSHR: follicle-stimulating hormone receptor. N: asparagine. S: serine. rFSH: recombinant follicle-stimulating hormone. uFSH: urinary FSH. * - for the difference between rFSH and uFSH, p>0.05. ** - for the difference between rFSH and uFSH, p<0.05.

#### *In vitro* data

**Figure 2:**
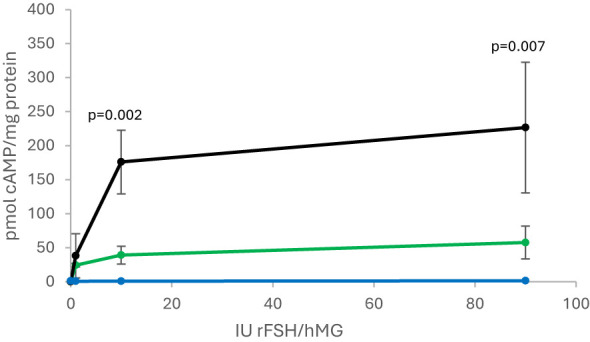
Cyclic-AMP measured from FSHR S-allele activity in response to 0, 1, 10, and 90 IU rFSH (green line) and uFSH (black line). The blue line represents the empty vector in response to rFSH and uFSH. FSHR, follicle-stimulating hormone receptor. rFSH, recombinant follicle-stimulating hormone; uFSH, human menopausal gonadotropin.

In COS-1 cells transfected with FSHR N680S variants, the S variant displayed higher extracellular cAMP per milligram of total protein when stimulated with uFSH compared to rFSH (10 IU: 176 vs. 39 pmol/mg, p=0.002; 90 IU: 227 vs. 58 pmol/mg, p=0.007; **Figure 2**). In COS-1 cells expressing the N variant, no statistically significant difference between the effect of uFSH and rFSH was observed.

### Clinical outcomes in genotyped and optimally treated women vs. non-genotyped controls

**Figure 3:**
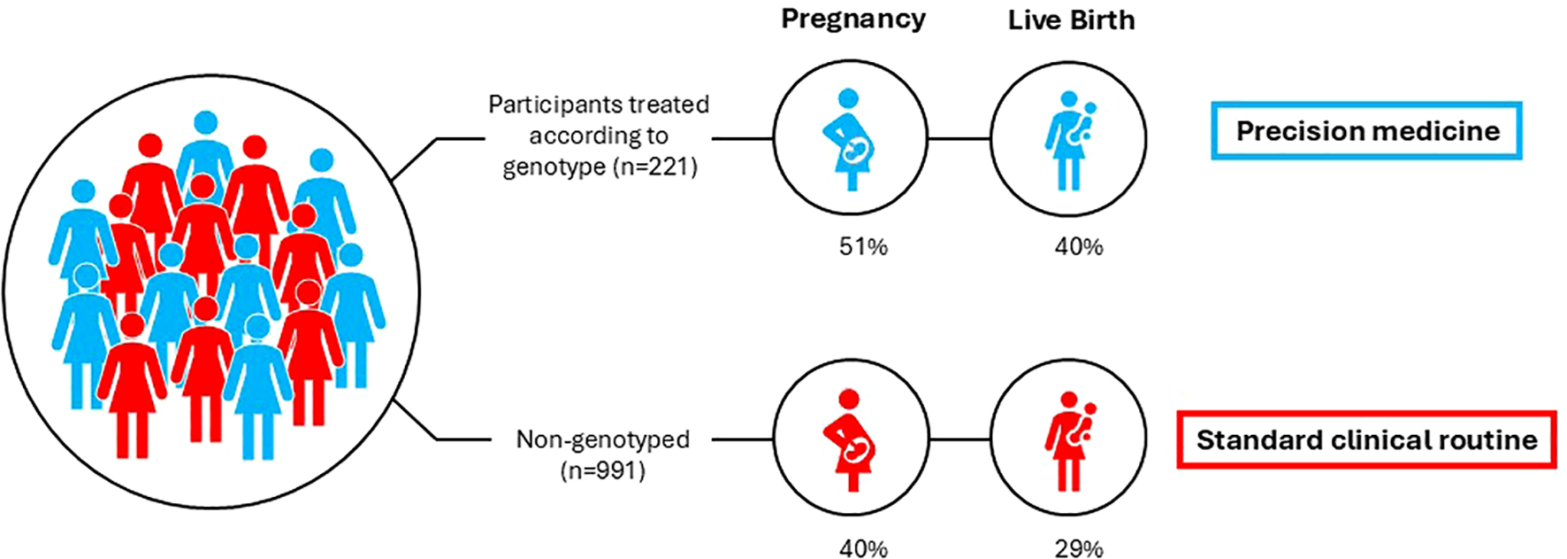
Chance of pregnancy and live birth in the genotyped (blue) and non-genotyped cohorts (red), respectively.

Because our findings presented above, both clinical and *in vitro*, were in favor of the hypothesis that S carriers would benefit from OS with uFSH, whereas some advantage of giving rFSH to NN carriers was seen in clinical settings, we defined the 160 women in the former and 61 in the latter group as a cohort of genotyped, optimally treated women, for a total of 221 (**Figure 1**). Compared to the 991 non-genotyped controls, in whom the choice of hormone was based on standard clinical criteria, the optimally treated cohort achieved higher cumulative pregnancy rates (51% vs. 40%; adjusted OR: 1.40, 95% CI 1.12–1.75, p=0.003) and higher CLBR (40% vs. 29%; adjusted OR: 1.55, 95% CI 1.23–1.96, p<0.001) in their first OS cycle (**Figure 3**, **Table 5**). The results were robust to adjustment for the fertilization method applied.

**Table 5:** Cumulative pregnancy rates and live birth rates for the genotyped population treated according to the FSHR N680S genotype and for the non-genotyped population treated according to clinical routine.

Population	Cumulative pregnancy rates, n (%)	Clinical live birth rates, n (%)
Genotyped population treated according to FSHR N680S genotype (n=221)	113 (51)*	89 (40)**
Non-genotyped population (n=991)	394 (40)*	290 (29)**

FSHR, follicle-stimulating hormone receptor. N, asparagine. S, serine. * - for difference between the genotype and the non-genotyped, p=0.003. ** - for difference between the genotype and the non-genotyped, p<0.001.

### Miscarriage and OHSS

The miscarriage rate in the genotyped and optimally treated cohort was 7%, whereas the corresponding proportion for controls was 10%. For severe OHSS, the frequency was 0.6% (all FSHR N680S NN and uFSH-treated) in the genotyped group.

## Discussion

In this clinical study, we found that genotyped women who were given uFSH if they carried the S allele, and rFSH if they were NN carriers, the CLBR in the first OS cycle was 40%. This was statistically significantly higher than the 29% observed in the non-genotyped controls treated according to current clinical routine and may be related to the fact that globally, even a 1% increase in live birth rates leads to thousands of additional births (21). When comparing the effect of uFSH and rFSH, both clinical and *in vitro* data pointed to the advantage of the urinary type in S allele carriers. These findings are in line with those previously reported by a smaller study by Lledo et al., 2016 (10), in which S-allele-carrying oocyte donors, serving as their own controls, responded to highly purified uFSH administration with higher oocyte numbers than when the same women were treated with rFSH. In addition, recipients of oocytes from S-allele-carrying donors displayed higher clinical pregnancy rates compared to NN-donors undergoing OS with uFSH (9). However, this difference was not statistically significant, probably due to a small sample size.

In cases with the NN genotype, the number of aspirated oocytes was 33% higher in those administered rFSH, which is consistent with a number of previous studies and also a previous meta-analysis on this topic (6). A positive association between the *per-cycle* number of aspirated oocytes and CLBR has previously been shown (22). However, in the current study, this subgroup was not large enough to show any statistically significant difference in cumulative pregnancy rates or CLBR. The biological mechanism behind these observations is still unknown, although the NN variant is considered to be more sensitive to rFSH than NS/SS. This phenomenon has not only been shown *in vitro* (8, 23), but also *in vivo* (24, 25). Human granulosa-lutein cells have also been demonstrated to be more sensitive to rFSH in cases with NN as compared to SS (23). Our finding of higher *in vitro* FSHR activity in cases with the S680 variant stimulated with uFSH does not indicate that the clinical advantage of using uFSH for S allele carriers is due to a longer half-life of this hormone, as previously suggested (10) but rather that the level of urine-derived hormones is somehow beneficial for women with this receptor constitution. Another possibility is the potential LH effect of uFSH. A review on this topic reported that LH supplementation is most beneficial in women with adequate prestimulation ovarian reserve parameters and an unexpected hyporesponse to rFSH monotherapy and in older women (36–39 years) undergoing treatment. There was no evidence that LH is beneficial in young (<35 years) normoresponders (26). Liu et al. suggested that the effect of uFSH may be due to promoting better oocyte and embryo quality and endometrial thickness compared with recombinant gonadotropins (27). In our study, NN women treated with uFSH did not differ from those receiving rFSH in terms of pregnancy and CLBR. Hence, if the effect is LH-driven, it does not seem to apply to women of all N680S genotypes.

In the context of applying precision medicine to OS prior to IVF, the most important finding was an 11% higher CLBR in the first OS cycle in women who had been treated with the type of FSH that best matched their FSHR N680S genotype as compared to non-genotyped controls. Our *post hoc* power analysis showed that with our sample size, we had 80% power to detect a 33% relative increase in CLBR in the former group. In our study, this relative increase was 38%, which is a significant difference. For couples undergoing assisted reproduction, the highest possible live birth rate is of the utmost importance when selecting an IVF clinic for treatment. Reported differences between IVF centers are frequently less than 5% (28). Hence, apart from being directly clinically applicable, our results indicate that taking the matter of pharmacogenetics into consideration when planning hormonal protocols for OS could also have health and economic implications by minimizing the number of repeated IVF cycles. By applying the simplified health economic evaluation proposed by Feng et al. (2024) (29), the implementation of genotyping at a cost of 1,000 USD would reduce the cost per live-born baby in the first IVF cycle from 41 380 USD to 31 000 USD.

To our knowledge, this is the largest study undertaken in which IVF outcomes were evaluated in relation to the FSHR N680S polymorphism and uFSH vs. rFSH. This represents a novel approach and may signify an important step toward precision medicine in the field of IVF. The comparison of a genotyped cohort who was administered FSH according to the recommendations given above, with a non-genotyped group, in whom the choice of hormone was based on traditional clinical criteria, clearly indicates the benefits of pre-treatment genotyping of all women undergoing assisted reproduction, egg freezing, or egg donation. A strength of the study is the use of live birth as an endpoint when comparing the outcomes in the genotyped vs. non-genotyped cohorts. The difference in pregnancy and live birth rates seen between the genotyped and optimally treated cohort and those non-genotyped is hardly due to selection bias, as the two groups did not differ significantly in terms of age, BMI, total hormone dose, or AMH concentrations. Another strength is the principle of using sealed envelopes to randomize participants to either rFSH or uFSH. Furthermore, to avoid bias, the clinicians who were prescribing medication were not involved in recruiting patients for the study, which was instead managed by a research nurse. The participants were recruited for their first OS cycle; therefore, no information from previous stimulations could influence the stimulation regimen of the recruited OS cycle. Furthermore, the FSHR N680S genotype was determined after hormone treatment to minimize bias.

Nevertheless, our study also had some shortcomings. The study was not sufficiently powered to evaluate the impact of our precision medicine concept on the risk of miscarriage or OHSS, nor can our results be extrapolated to the categories of women who were excluded, e.g. those with PCOS, endometriosis, etc. It remains to be seen whether these women, in whom the outcome of IVF may be negatively affected by the aforementioned pathological conditions, would benefit from a choice of FSH in relation to the N680S variants. Future studies to provide comprehensive evidence are warranted. Furthermore, all rFSH-treated women were given follitropin alpha. However, there is no evidence that follitropin alpha is inferior to the other rFSH variants—beta and delta (30–32). Furthermore, no difference in pregnancy and live birth rate was shown when Menopur® was applied as uFSH in the current study compared to another uFSH (33). We therefore believe that the results can be generalized to all rFSH and uFSH subtypes. However, a conclusion remains to be drawn from future studies to confirm the generalizability of this approach.

Regarding the applicability of the results to other ethnicities, and comparing the Caucasian distribution to other ethnic groups, the distribution is different, with the ancestral N allele having a low allele frequency in sub-Saharan Africans, whereas the S allele is enriched in Europeans, the Middle East, Central South Asia, and Oceania, with its lowest frequency in Far East Asia and North America (34). Although no data on the impact of ethnicity on the interaction between the FSHR N680S genotype and FSH type are available, an increased risk of poor response in SS carriers in Asian women has been reported (35). Additionally, Polyzos et al. found an impact of FSHR N680S on the outcome of OS even after adjusting for European vs. Asian origin (7). These findings may indicate that ethnicity is not a crucial factor in determining the impact of the N680S polymorphism on OS outcome.

In summary, a significantly increased chance of pregnancy and live birth can be achieved by an FSHR genotype-guided approach. While the administration of uFSH would be the choice for S carriers, rFSH would be beneficial for NN-carrying women. An individually adapted, and thus also more efficient, treatment would not only be a completely new strategy but also cut costs, as the number of repeated, expensive, and potentially challenging treatments could presumably be reduced. However, the current study only comprises women undergoing their first OS cycle. Other underlying causes of response failure may play a role in those treated multiple times. In these women, genotyping would have limited value.

## Conclusions

To date, there is no clinically useful, reliable marker for the outcome of an IVF treatment. Thus, different gonadotropin preparations at different doses are evaluated empirically. Consequently, many women experience repeated and often challenging hormonal stimulation cycles. With this background knowledge, our scientific question was: Is it beneficial to genotype women for the FSHR N680S variant to guide the choice of gonadotropin prior to OS? Our study evidently shows that this genetic variant can be utilized in the choice of hormone type prior to IVF. If the correct hormone according to the individual’s FSHR N680S genotype was administered, the relative chance of having a baby increased by 38% in the first cycle compared to non-genotyped, but otherwise matched controls. Hence, with a genotype-guided approach to gonadotropin choice in OS of women not diagnosed with PCOS or endometriosis, the success rate in terms of live births would increase significantly, whereas unnecessary hormone stimulations would decrease. Future studies should include other diagnostic groups than those encompassed by the current report, test other FSH types, and explore the impact of population diversity on the findings reported by us.

## Data Availability

The data presented in the study are deposited in the SND (Swedish national data services) repository, accession nummber SND-ID: 2025-49. The data is under review, but the preview of the catalogue entry can be found here: https://researchdata.se/en/catalogue/dataset/2025-49/1?previewToken=e3ed3212-cef6-4472-a2de-7c19cd528a68.
